# Integration of single‐cell and RNA‐seq data to explore the role of focal adhesion‐related genes in osteoporosis

**DOI:** 10.1111/jcmm.18271

**Published:** 2024-03-27

**Authors:** Xiaojian Shi, Qiang Fu, Jianyu Mao, Jiajie Yang, Ye Chen, Jiajia Lu, Aimin Chen, Nan Lu

**Affiliations:** ^1^ Department of Orthopedic Trauma Surgery Haimen People's Hospital Nantong Jiangsu China; ^2^ Department of Orthopedic Trauma Surgery Shanghai Changzheng Hospital Shanghai China; ^3^ Department of Orthopedic Trauma Surgery Shanghai Fourth People’s Hospital, School of Medicine, Tongji University Shanghai China

**Keywords:** bulk RNA‐sequencing, focal adhesion, osteoporosis, single‐cell RNA sequencing

## Abstract

Integrin‐based focal adhesion is one of the major mechanosensory in osteocytes. The aim of this study was to mine the hub genes associated with focal adhesion and investigate their roles in osteoporosis based on the data of single‐cell RNA sequencing and RNA‐sequencing. Two hub genes (FAM129A and RNF24) with the same expression trend and AUC values greater than 0.7 in both GSE56815 and GSE56116 cohorts were uncovered. The nomogram was created to predict the risk of OP based on two hub genes. Subsequently, the competing endogenous RNA network was established based on two hub genes, 14 microRNAs and five long noncoding RNAs. Meanwhile, transcription factors‐hub gene network was established based on two hub genes and 14 TFs. Finally, 73 drugs were predicted, of which there were 13 drugs targeting FAM129A and 66 drugs targeting RNF24. In both mouse and human blood samples, FAM129A expression was decreased in granulocytes and RNF24 expression was increased in monocytes. In the mouse experiment, FAM129A and anti‐RNF24 were found to partially alleviate the progression of osteoporosis. In conclusion, two hub genes related to focal adhesion were identified by combined scRNA‐seq and RNA‐seq analyses, which might supply a new insight for the treatment and evaluation of OP.

## 1 INTRODUCTION

Osteoporosis (OP) is a disease characterized by loss of bone mass, destruction of bone tissue microstructure and decreased bone strength. OP is a common outcome of the ageing population with more than 200 million sufferers worldwide, and is the third most prevalent disease among older people.^1^ The diagnosis of OP is mainly based on bone mineral density examination and imaging examination, combined with clinical symptoms. Imaging examination can observe the changes of bone morphology and structure.^2^ Early diagnosis and treatment of OP are very important to reduce the risk of fracture and improve the quality of life of patients. However, there is a lack of highly sensitive and accurate methods for early diagnosis. Therefore, in‐depth research to identify potential diagnostic genes will contribute to our understanding of OP disease progression and treatment.^3^, ^4^

The progression of OP is often accompanied by the aggregation of granulocytes and monocytes. Therefore, focal adhesion genes of cells are one of the areas of interest.^5^ Focal adhesions are specialized structures at the contact points of cellular‐extracellular matrix, where bundles of actin filaments are anchored to transmembrane receptors of the integrin family through a multimolecular complex of junctional plaque proteins. Some constituents of FA genes participate in the structural link between membrane receptors and the actin cytoskeleton, while others are signalling molecules.^6^ Studies have shown that the role of focal adhesion in the regulation of bone metabolism is mainly achieved through cell–matrix interaction.^7^ In addition, it was found that the incidence of OP could be reduced by regulating the composition and activity of focal adhesion.^8^ For example, some drugs can modulate focal adhesion of osteocytes by affecting the expression and function of focal adhesion components such as integrins, thereby slowing the rate of bone loss and promoting bone building.

Single‐cell sequencing (scRNA‐seq) is a high‐throughput genomic technology that can simultaneously determine the entire gene expression information of a single cell, so as to gain insights into gene expression patterns, cell states and functions of different cell types.^9^ Single‐cell sequencing at different time points or different bone tissues can reveal the changes and transformation of different cell subsets during the development of OP, discover key genes and biological events, and then explore the development mechanism of the disease and potential therapeutic targets and programs.^10^, ^11^ Some drugs can regulate focal adhesion of osteocytes by affecting the expression and function of focal adhesion components such as integrins, thereby slowing the rate of bone loss and promoting bone building. At present, the role of focal adhesion in OP needs to be further studied.

Hence, the aim of this study was to mine the hub genes associated with focal adhesion and investigate their roles in OP based on the data of scRNA‐seqand bulk RNA‐seq. Two hub genes (FAM129A and RNF24) related to focal adhesion were identified by combined scRNA‐seq and bulk RNA‐seq analyses. Moreover, in both human and mouse studies, we validated the significant differences between granulocytes and monocytes in the progression of OP.

## 2 MATERIALS AND METHODS

### 2.1 Data source

The GSE147287, GSE56815 and GSE56116 datasets were acquired from the Gene Expression Omnibus (GEO) database (http://www.ncbi.nlm.nih.gov/geo/). The GSE147287 cohort included the data of single‐cell RNA sequencing (scRNA‐seq) of one osteoporosis (OP) specimens and one osteoarthritis specimens. The GSE56815 cohort (GPL96, monocytes) included the RNA sequencing (RNA‐seq) data of 20 OP specimens and 20 normal control (NC) specimens. GSE56116 cohort (GPL4133, peripheral blood) included the RNA‐seq data of 10 OP specimens and three NC specimens. GSE56815 cohort was utilized as the training set and GSE56116 cohort was utilized as the validation set. Besides, 199 focal adhesion‐related genes (FARGs) were collected from Msigdb database (https://www.gsea‐msigdb.org/gsea/msigdb) (Table S1).

### 2.2 scRNA‐seq analysis

The scRNA‐seq data was filtered by Seurat package (v4.1.0) (min. cells = 3, min. features = 200).^12^ The criteria for quality control (QC) was nFeature RNA >100 and <5000, nCount RNA <20,000 and percent.mt <5%. Then, the data were normalized using Normalize Data function, and Find Variable Features function was used to identify genes with high variation in expression among cells. After that, the data were uniformizated using Scale Data function and subjected to principal component analysis (PCA). The contribution of each principal component (PC) to the variation was shown via Elbow Plot function. To identify the availability of data, Jack Straw and Score Jack Straw were utilized to assess the significance of every gene in each PC. After PCA finished, unsupervised cluster analysis on the data was completed through the Seurat package (resolution = 0.2). Subsequently, the annotation of cell types was completed through the SingleR package (v1.8.0)^13^ based on the Human Primary Cell Atlas Data in celldex package (v1.4.0).^13^ In addition, the marker genes of each type of cells were retrieved via Find‐All‐markers function (min.pct = 0.2, only.pos = TRUE). The proportion of different cell types in two specimens in GSE147287 cohort was displayed in the histogram. Fisher's test was utilized to mine key cells depending on the distribution of different cells in OP and osteoarthritis specimens (odds ratio (OR) >5, *p* < 0.05). Additionally, cell communication analysis of the annotated cell types was performed by CellChat package (v1.4.0).^14^ Similarly, UMAP dimensionality reduction and cell clustering analysis were completed to identify the subtypes of key cells via Seurat package (resolution = 0.2). The marker genes of different cell subtypes were obtained via Find‐All‐markers function (min.pct = 0.2, only.pos = TRUE). Finally, Fisher's test was utilized to explore the distribution of different cell subtypes in OP and osteoarthritis specimens (OR >5, *p* < 0.05).

### 2.3 Functional enrichment analysis

Gene Ontology (GO) and Kyoto Encyclopedia of Genes and Genomes (KEGG) analyses were implemented via cluster Profiler package (v4.2.2) (*p*.adj <0.05).^15^

### 2.4 Differential expression analysis in GSE56815 dataset

The limma package (v3.50.1)^16^ was utilized to extract the differentially expressed genes (DEGs) between OP specimens and NC specimens in GSE56815 dataset (*p*.adj <0.05).

### 2.5 Weighted gene coexpression network analysis (WGCNA)

The grouping of OP and NC was used as clinical traits, and the WGCNA package (v1.70.3)^17^ was used to identify OP‐associated genes. Firstly, to ensure the accuracy of the subsequent analysis, the specimens were clustered to remove the outliers. Then, the optimal soft threshold was determined to ensure the network approached scale‐free distribution. The coexpression modules were obtained from the scale‐free network constructed based on the optimal soft threshold. Subsequently, the correlation analysis was utilized to find key modules associated with OP.

### 2.6 Identification of candidate hub genes and protein–protein interaction (PPI) network creation

The common marker genes were filtered by overlapping the marker genes of key cell and the marker genes of key subpopulation of key cell. The differentially expressed FARGs (DE‐FARGs) were obtained by overlapping the DEGs between OP and NC specimens, FARGs and module genes from WGCNA. Spearman correlation analysis was utilized to mine the relevance of DE‐FARGs to common marker genes. The common marker genes of top 30 with high correlation to DE‐FARGs were screened as candidate hub genes for subsequent analysis. The PPI network was implemented through STRING database (https://string‐db.org/) (medium confidence >0.4).

### 2.7 Construction of diagnostic models and nomogram

Univariate logistic regression and least absolute shrinkage and selection operator (LASSO) regression analyses were utilized to further mine hub genes. The univariate logistic regression analysis was implemented via glm function. The LASSO regression analysis was performed via glmnet package (v4.1‐2).^18^ The pROC package (v1.18.0) was utilized to paint receiver operating characteristic (ROC) curve.^18^, ^19^ The nomogram was established via rms package (v6.2‐0).^20^ The decision curve and clinical impact curve were plotted to evaluate the clinical predictive value of the nomogram via rmda package (v1.6) (https://www.rdocumentation.org/packages/rmda).

### 2.8 Gene set enrichment analysis (GSEA)

Firstly, specimens were divided into high‐ and low‐expression groups based on the expression of hub genes. Then, differential expression analysis between high‐ and low‐expression groups was performed using limma package (v3.50.1)^16^ in the training set, and logFC was calculated. After sorting the logFC, single‐gene GSEA enrichment analysis was implemented depending on the KEGG gene sets in MSigDB database (https://www.gsea‐msigdb.org/gsea/msigdb/) (*p* < 0.05).

### 2.9 Drug prediction based on the hub genes

The therapeutic drugs targeting hub genes were mined via CTD database (https://www.ctdbase.com/). The drug‐hub gene network was established via Cytoscape software (v3.8.2).^21^

### 2.10 Construction of regulatory network based on hub genes

The microRNAs (miRNAs) targeting hub genes were mined by miRDB database (https://mirdb.org/) (target score >80). The long noncoding RNAs (lncRNAs) targeting miRNAs were mined from Starbase database (https://starbase.sysu.edu.cn/index.php) (low stringency ≥1). The transcription factors (TF) targeting hub genes were predicted via Network Analyst database (https://www.networkanalyst.ca/). The competing endogenous RNA (ceRNA) network and TF‐hub gene network were created via Cytoscape software (v3.8.2).^21^

### 2.11 The expression of hub genes in cells

The expression of hub genes in clustered cells and subpopulations of key cells was demonstrated by plotting bubble and clustering maps. Then, the differentiation trajectories of all cells and key cells were simulated using the monocle package (v2.22.0).^22^

### 2.12 Animal experiments

Animal experiments were carried out in accordance with the guidelines of the Medical Ethics Committee of Shanghai Changzheng Hospital. Eight‐week‐old female C57BL/6J mice were randomly divided into four groups (*n* = 5/group). The sample number was based on a preliminary experiment (90% confidence interval, 5% type I error risk and 10% type II error risk): the sham+PBS group (no operation, but exposure of bilateral ovaries, retained intact and injection of 0.1 mL PBS/3 d); OVX+PBS group (bilateral ovariectomy, and injection of 0.1 mL PBS/3 d); and OVX+ani‐RNF 24 group (bilateral ovariectomy, 0.1 mL injection of anti‐RNF24/3d, 100 mg/kg, R&D Systems); OVX + FAM129a group (bilateral ovariectomy, and injection of 0.1 mL FAM129A/3d, 100 mg/kg, US Biological). Eight weeks later, three mice were randomly selected from each group for the following experiments: (a) The serum was separated by centrifugation from blood taken from the tail vein and was detected and analysed using an ELISA (Bio Legend); (b) microscopic CT scanning was performed on bilateral femur. The remaining mice in each group (*n* = 2) were used for cell experiments.

### 2.13 Human serum experiments

All patients and subjects were informed before their inclusion, and written consent was given. The experimental protocols were approved by the Medical Ethics Committee of Shanghai Changzheng Hospital. Six OP patients (five females and one male) and six nonosteoporosis volunteers (five females and one male) provided blood samples which were separated by centrifugation (Histopaque‐1077 and Histopaque‐1119, Sigma‐Aldrich) to obtain granulocytes and monocytes. qRT‐PCR experiment was performed to obtain the changes of relative genes.

### 2.14 Statistical analysis

All open databases and R software (v4.1.0) were utilized to analyse and visualize in this study. The Venn diagram was plotted via Venn Diagram package (v1.7.1).^23^ If not specified above, a *p*‐value less than 0.05 was considered statistically significant.

## 3 RESULTS

### 3.1 Identification of key cells and their marker genes in GSE147287 cohort

**FIGURE 1:**
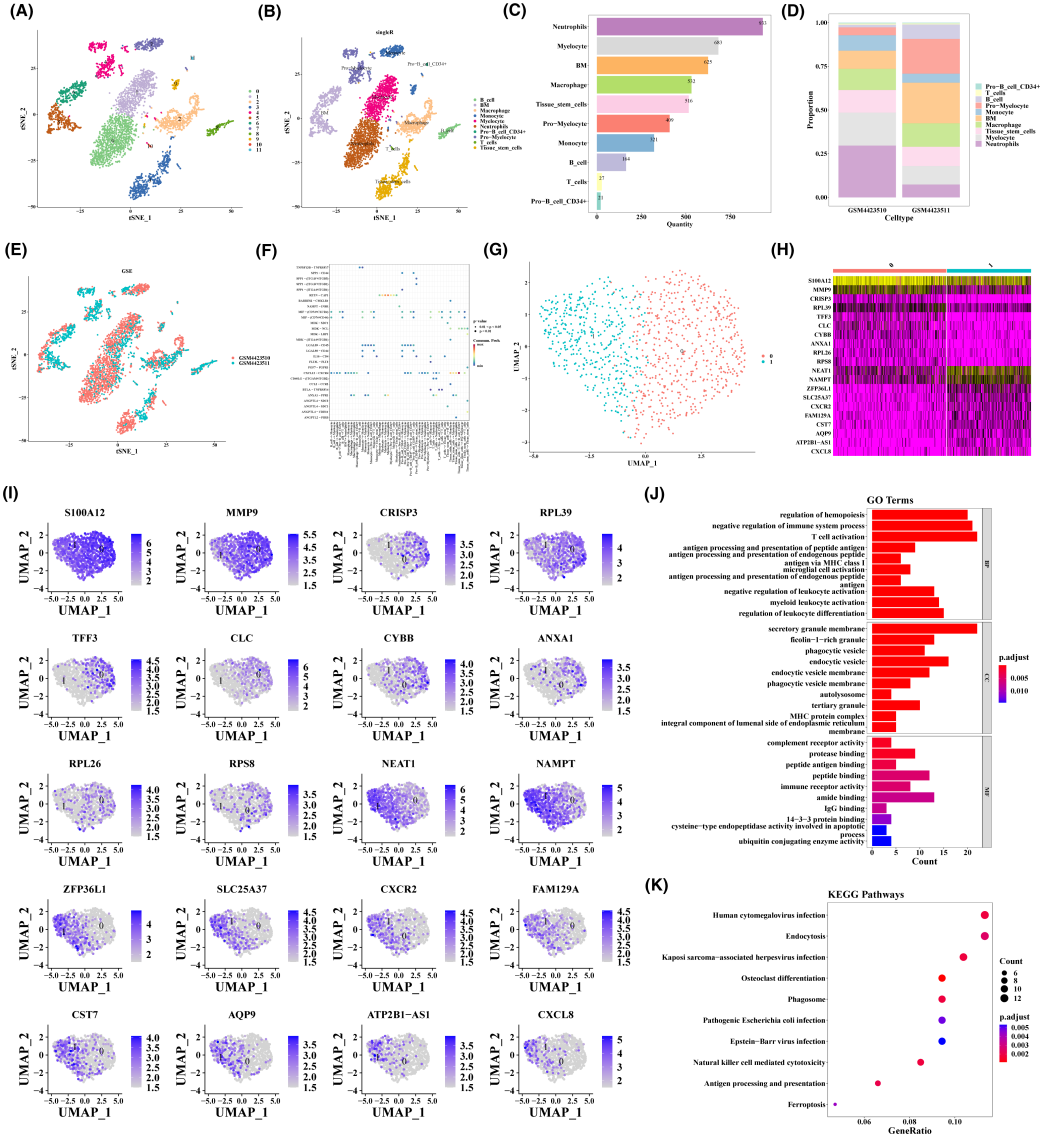
Dimensionality reduction clustering and annotation of single‐cell sequencing data to identify key cells. And identification of key cell subtypes of neutrophils. (A) In total, 12 cell groups were obtained via TSNE clustering analysis (the resolution was 0.2). (B) 10 cell subpopulations including B cells, BM, macrophages, monocytes, myelocytes, neutrophils, pro‐B cells CD34+, pro‐myelocytes, T cells and tissue stem cells were annotated via SingleR package. (C) Bar graphs are used to present the number of 10 cell types in the sample. (D) Histograms were drawn to show the proportion of different cell types in GSM4423510 and GSM4423511. (E) The TSNE clustering was visualized according to the groups in GSM4423510 and GSM4423511. (F) The bubble diagram showed the probability of communication between cells for different ligands and receptors. (G) UMAP dimension reduction analysis and cell clustering analysis were performed on Neutrophils (resolution = 0.2). (H) The top10 marker genes in each cell subtype were selected to draw the expression heat map (FindAllmarkers, min.pct = 0.2，only.pos = TRUE). (I) UMAP cluster map of top10 marker genes for each cell subtype. (J) Visualization of GO enrichment results for Neutrophil 1 marker genes (The length of the cylinder represents the number of genes enriched to the biological function.). (K) Visualization of KEGG enrichment results for Neutrophil 1 marker genes.

After the data filtering was completed, 18,187 cells and 20,955 genes met the criteria (Figure S1A). Figure S1B demonstrated the 4231 cells and 20,955 genes were acquired after QC. Then, top 2000 highly variable genes were selected for cell type identification, and the top 10 genes were labelled (Figure S1C). After that, the data were normalized, and the result of PCA was shown in Figure S1D. Then, top 25 PCs were selected for subsequent analysis (*p* < 0.05) (Figure S1E,F). The resolution was confirmed before clustering, and clusters worked well when the resolution was 0.2. In total, 12 cell groups were obtained via TSNE clustering analysis (Figure 1A). Subsequently, 10 cell subpopulations including B cells, BM, macrophages, monocytes, myelocytes, neutrophils CD34+, pro‐B cells, pro‐myelocytes, T cells and tissue stem cells were annotated via SingleR package (Figure 1B). Figure 1C revealed that there were 933 neutrophils, which was the most numerous cell type. The expression of top 10 marker genes in 10 cell subpopulations for each cell type was shown in Figure S2. The proportion of different cell types in OP and osteoarthritis specimens was further mined. The results revealed that the neutrophil was the main cell type in the OP specimens, and the BM was the main cell type in the osteoarthritis specimens (Figure 1D). Figure 1E showed the distribution of cells in the two samples. Subsequently, neutrophils which including 382 marker genes were defined as key cells according to Fisher's test (OR >5, *p* < 0.05) (Table S2). Then, we investigated the number and strength of interactions among 10 cell subpopulations (Figure S3A,B). The bubble diagram showed the probability of communication between cells for different ligands and receptors, where CXCL12‐CXCR4 had more probability (Figure 1F).

### 3.2 Identification of subtypes of key cells

Firstly, we performed UMAP dimensionality reduction analysis and cell clustering analysis on neutrophils based on PC1‐PC25. A total of two different cell subtypes of neutrophils (neutrophils‐0 and neutrophils‐1) were identified (Figure 1G). Then, the distribution of top10 marker genes in two cell subtypes were demonstrated in Figure 1H,I. According to Fisher's test, neutrophil‐1, which contained 184 marker genes, was recognized as a key cell subtype (Table S3). After that, we performed a functional enrichment analysis based on 184 marker genes. GO results revealed that these marker genes were associated with immune‐related pathways such as T cell activation, negative regulation of leukocyte activation, negative regulation of immune system process, etc. (Figure 1J). As for KEGG, osteoclast differentiation, phagosome, antigen processing and presentation, ferroptosis were enriched in these marker genes (Figure 1K). The above‐mentioned pathways were associated with OP and osteoarthritis.

### 3.3 Identification of OP‐related key modules by WGCNA

**FIGURE 2:**
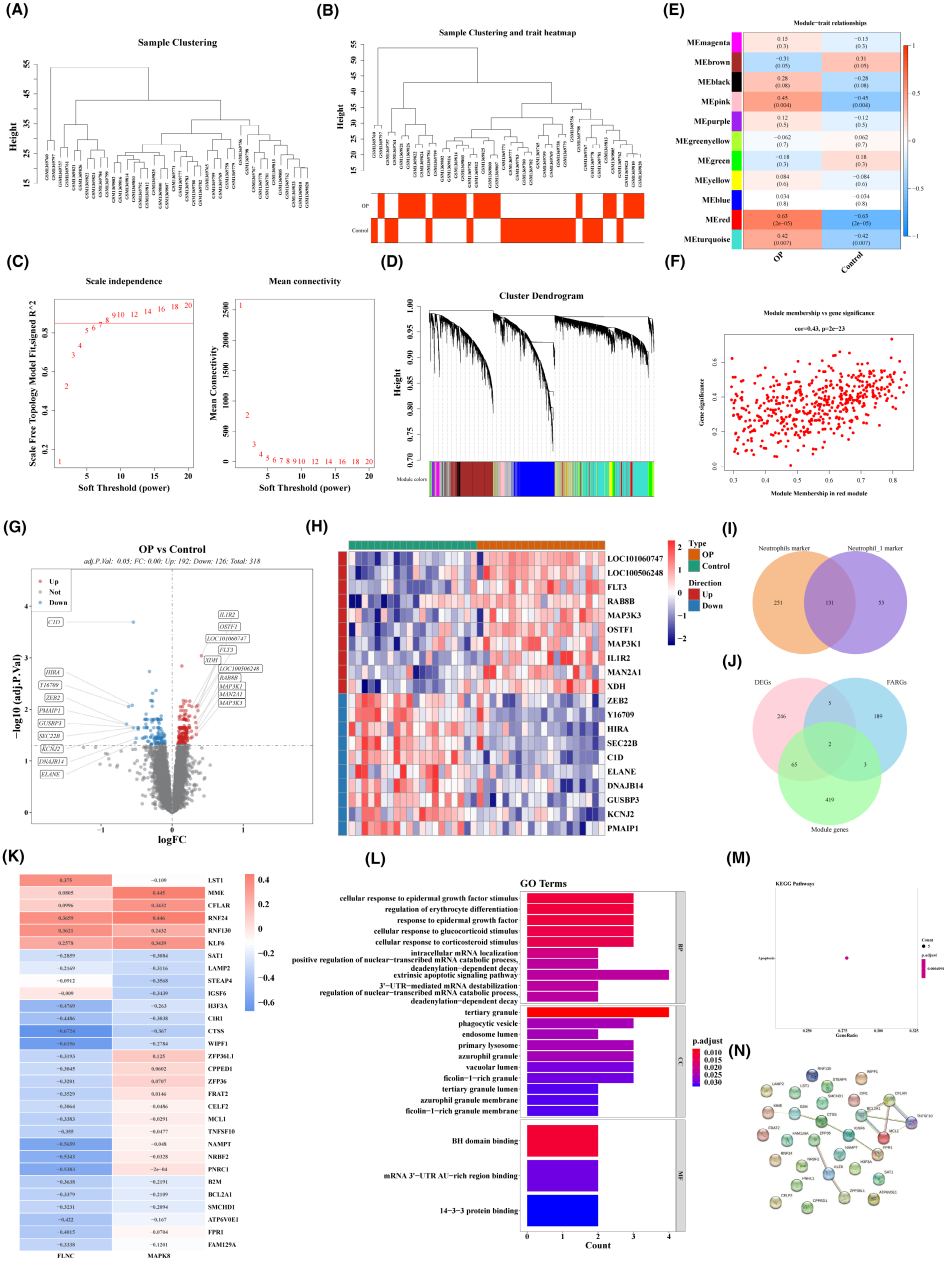
WGCNA analysis was used to identify gene modules with high correlation with OP and identification of candidate hub genes and function analysis. (A) Sample hierarchical clustering. (B) Sample hierarchical clustering and heat map after clinical traits. (C) Soft threshold screening. (D) Identification of coexpression modules. (E) Heatmap of correlation of modules and phenotypes. (F) Scatter plot of correlation between modules and genes. (G) Volcano plot of differentially expressed genes between OP and Control. (H) The heat map of differentially expressed genes (top10 upregulated genes and downregulated genes) between OP and Control. (I) Identification of intersection marker genes. (J) Identification of differential focal adhesion‐related genes. (K) Heat map of correlation between candidate genes and differential focal adhesion‐related genes. (L) Visualization of GO enrichment results for candidate genes. (M) KEGG enrichment results of candidate genes were visualized. (N) PPI network of candidate hub genes.

To select the modules and genes most associated with OP, we performed WGCNA analysis in GSE56815 cohort. As shown in Figure 2A, there was no outlier sample. The sample clustering and heat map of clinical traits were shown in Figure 2B. Moreover, when the soft threshold (power) was 8, *R* ^2^ was 0.85, wihch meant connectivity tend to 0 (Figure 2C). Then, 11 modules were obtained by dynamic tree cutting algorithm (Figure 2D). Subsequently, the red module containing 489 genes was selected as OP‐related module based on the results of module‐trait correlation analysis (Figure 2E). The relevance of gene significance (GS) and module membership (MM) in the red module was shown in Figure 2F.

### 3.4 Identification of candidate hub genes and function analysis

In total, 318 DEGs were obtained between OP and NC specimens in GSE56815 cohort, with 126 downregulated genes and 192 upregulated genes in OP specimens (Figure 2G). The top 10 up‐ and down‐regulated DEGs were displayed in Figure 2H. Then, 131 common marker genes were filtered by overlapping 382 marker genes in neutrophils and 184 marker genes in neutrophil‐1 (Figure 2I). Meanwhile, two DE‐FARGs (*FLNC* and *MAPK8*) were acquired by overlapping 318 DEGs between OP and NC specimens, 489 key genes from WGCNA and 199 FARGs (Figure 2J). Then, 30 candidate hub genes were authenticated based on the relevance analysis between 2 DE‐FARGs and 131 common marker genes (Table S4). All candidate hub genes were correlated with DE‐FARGs with absolute values greater than 0.3, with the highest correlation between *CTSS* and *FLNC* (cor = −0.6724) (Figure 2K). To investigate the potential function of candidate hub genes, we performed GO and KEGG enrichment analyses. The results of GO revealed that 30 genes were correlated with regulation of erythrocyte differentiation and cellular response to epidermal growth factor, glucocorticoid stimulus and corticosteroid stimulus (Figure 2L). In KEGG, apoptosis was enriched in candidate hub genes (Figure 2M). Finally, the PPI network of 30 candidate hub genes was established to investigate the interactions among genes (Figure 2N).

### 3.5 Identification of hub genes and nomogram creation

**FIGURE 3:**
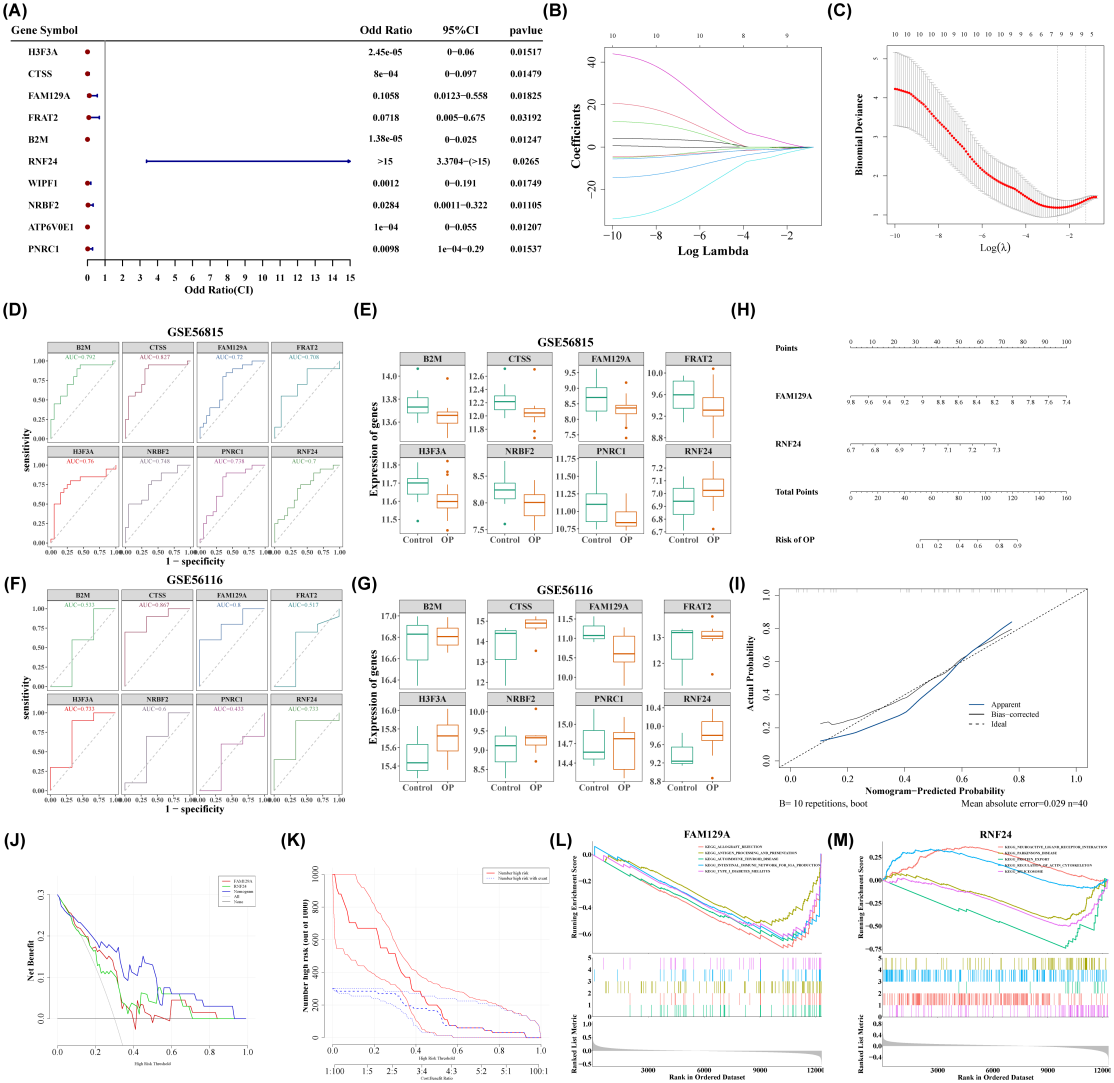
Screening of hub genes based on candidate hub genes and nomogram model construction and GSEA analysis of key genes. (A) The forest plot of univariate logistic analysis. (B) Spectrum of LASSO coefficients. (C) Ten‐fold cross‐validation of the adjusted parameters in LASSO analysis. (D) ROC curves of feature genes (GSE56815). (E) Boxplots of feature gene expression between OP and Control (GSE56815). (F) ROC curves of feature genes (GSE56116). (G) Boxplots of feature gene expression between OP and Control (GSE56116). (H) Construction of a nomogram model according to key genes. (I) The calibration curve for the nomogram model. (J) The decision curve for the nomogram model. (K) The clinical impact curve for the nomogram model. (L) GSEA enrichment analysis of FAM129A single gene. (M) GSEA enrichment analysis of RNF24 single gene.

In total, 10 genes (*H3F3A*, *CTSS*, *FAM129A*, *FRAT2*, *B2M*, *RNF24*, *WIPF1*, *NRBF2*, *ATP6V0E1* and *PNRC1*) were acquired via univariate logistic regression analysis (Figure 3A). Then, eight genes (*H3F3A*, *CTSS*, *FAM129A*, *FRAT2*, *B2M*, *RNF24*, *NRBF2* and *PNRC1*) were further tapped via LASSO regression analysis (Figure 3B,C). The AUC value of above eight genes was more than or equal to 0.7, indicating the diagnostic value was decent (Figure 3D). Moreover, *H3F3A*, *CTSS*, *FAM129A*, *FRAT2*, *B2M*, *NRBF2* and *PNRC1* were lowly expressed in the OP specimens, while *RNF24* was highly expressed (Figure 3E). Meanwhile, we also plotted ROC curves and performed expression analysis in GSE56116 cohort. Figure 3F revealed that the AUC value of *H3F3A*, *CTSS*, *FAM129A* and *RNF24* was all greater than 0.7, indicating a tolerable predictive power. Figure 3G revealed that *B2M*, *FAM129A* and *FRAT2* were less expressed in OP samples than in NC samples, while *CTSS*, *H3F3A*, *NRBF2*, *PNRC1* and *RNF24* were the opposite. In summary, *FAM129A* and *RNF24* with the same expression trend and AUC values greater than 0.7 in both GSE56815 and GSE56116 cohorts were treated as hub genes. Subsequently, the nomogram was created based on two hub genes to predict the risk of OP (Figure 3H). The calibration curve implied the effectiveness of nomogram (Figure 3I). The decision curve and clinical impact curve demonstrated the clinical applicability of the nomogram (Figure 3J,K).

### 3.6 Exploring the molecular mechanism of OP based on hub genes

To further investigate the potential function of hub genes, we performed GSEA analysis. The results revealed *FAM129A* was associated with PPAR signalling pathway, antigen processing and presentation, type I diabetes, and haematopoietic cell lineage and so on (Figure 3L, Table S5). Meanwhile, *RNF24* was correlated with ribosome, leukocyte transendothelial migeration and protein export etc (Figure 3M, Table S6). According to IPA analysis, hub genes were linked with cellular compromise, cellular function and maintenance and immunological disease (Figure S4A). As shown in Figure S4B, 21 upstream regulators were involved in the regulation of two hub genes.

### 3.7 The regulator network of ceRNA and TF‐hub gene

**FIGURE 4:**
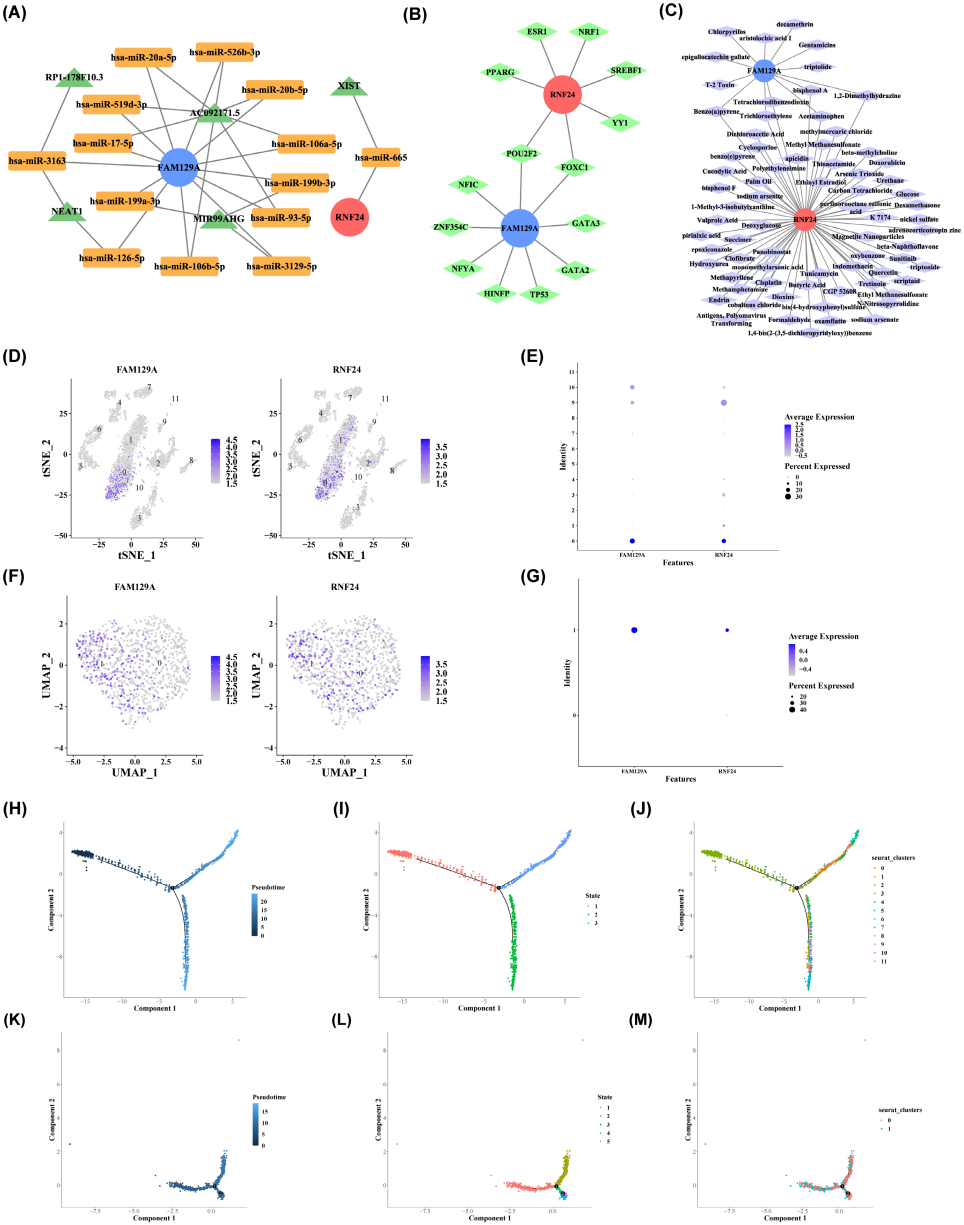
The regulatory network of hub genes and potential therapeutic drugs for OP and the expression of hub genes in cells. (A) The ceRNA regulatory network. (B) The TF‐hub genes regulatory network. (C) Potential therapeutic drugs targeting hub genes for OP. (D) Cluster map of the expression distribution of two hub genes in the 11 cell groups. (E) Bubble plot of the expression of two hub genes in the two key cell groups. (F) Cluster map of the distribution of two hub genes in the two key cell groups. (G) Bubble plot of the distribution of two hub genes in the two key cell groups. (H) Cell differentiation trajectories of all cells. (I) Three stages of cell differentiation trajectories. (J) Differentiation of 11 cell groups. (K) Cell differentiation trajectories of neutrophils. (L) Five stages of differentiation of the neutrophils. (M) Differentiation of two cell groups.

To explore the regulatory relationships of hub genes in organisms, we constructed the network based on hub genes, miRNAs, lncRNAs and TFs. Firstly, 14 miRNAs targeting two hub genes were mined from miRDB database. Then, five lncRNAs targeting miRNAs were obtained from Starbase database. The ceRNA network containing 21 nodes and 29 edges was established (Figure 4A). Figure 4A revealed that AC092171.5 could bind to hsa‐miR‐93‐5p, hsa‐miR‐526b‐3p and hsa‐miR‐519d‐3p, which in turn regulated the expression of *FAM129A*. Then, 14 TFs targeting two hub genes were acquired from Network Analyst database. The regulatory network of TF‐hub gene revealed that *FAM129A* and *RNF24* could be regulated by POU2F2 and FOXC1 (Figure 4B).

### 3.8 Prediction of potential therapeutic drugs for OP

To explore effective therapeutic drugs for OP patients, we performed drug prediction analysis. In total, 73 drugs targeting two hub genes were extracted in CTD database. The drug‐gene network (75 nodes and 79 edges) manifested that there were 13 drugs targeting *FAM129A* and 66 drugs targeting *RNF24* (Figure 4C). Figure 4C revealed that benzo(a)pyrene, bisphenol A and acetaminophen could act on two hub genes.

### 3.9 Analysis of hub genes in cells

To further investigate the mechanism of action of hub genes in OP, we mined the distribution of hub genes in cells. The distribution and expression of two hub genes in 11 cell groups were shown in Figure 4D,E. Figure 4F,G revealed the expression of *FAM129A* was higher than *RNF24* in two key cell subpopulations. The analysis of the differentiation trajectories of all cells showed there were three different states of cell differentiation (Figure 4H,I). As shown in Figure 4I, state 1 was the earliest stage of cell differentiation. Figure 4J represented the stages of differentiation of 11 cell groups. Figure S5A demonstrated *FAM129A* and *RNF24* were mainly expressed at state 2 and state 3. Meanwhile, we also simulated the differentiation trajectory of neutrophils. Figure 4K–M revealed that neutrophils had five stages of differentiation, and the earliest stage of differentiation was at state 1. Figure S5B displayed the expression of hub genes during the differentiation of neutrophils.

### 3.10 Analysis of human and mouse specimens

**FIGURE 5:**
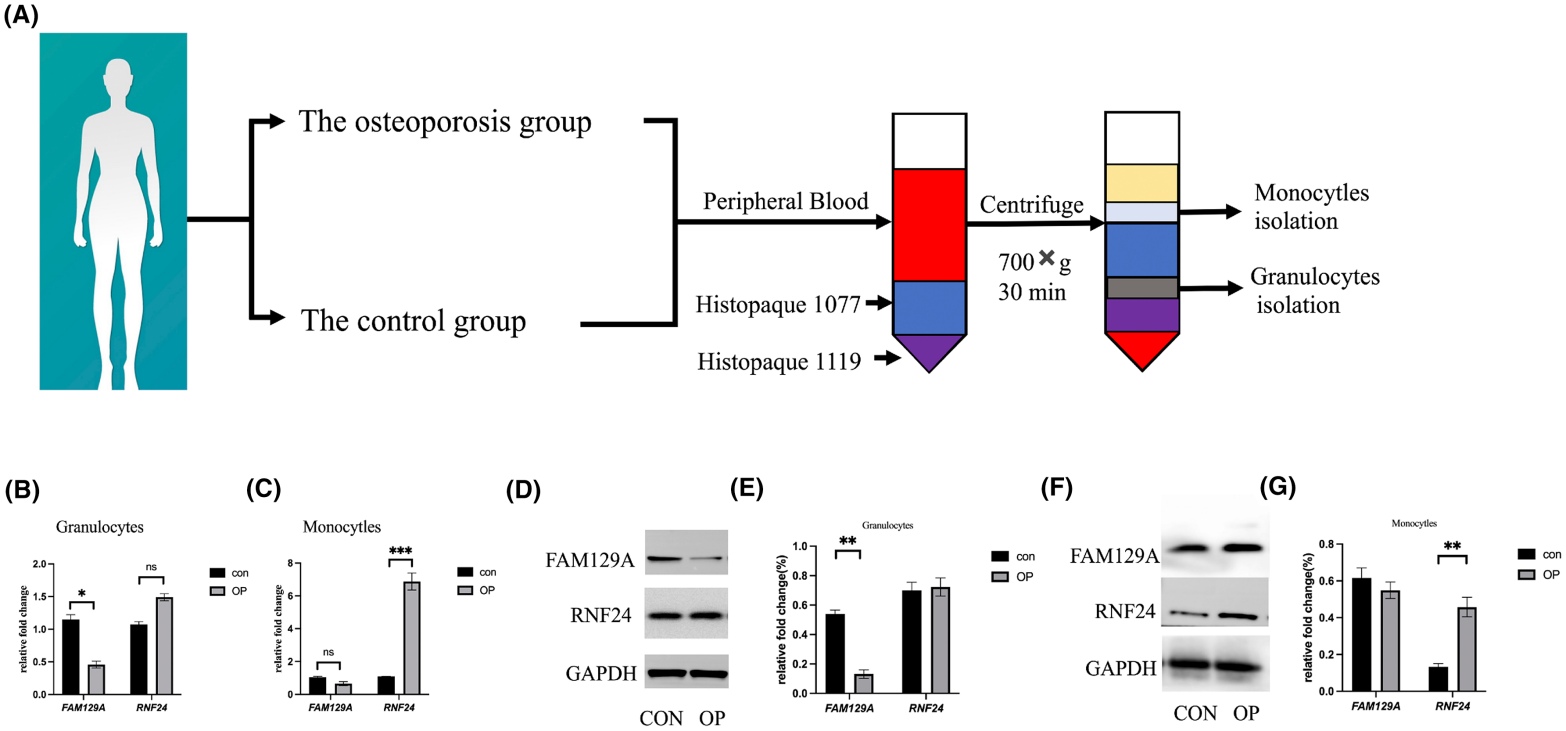
Analysis of the expression of FAM129A and RNF24 via human specimens. (A) Obtaining of different cells from the peripheral blood of two groups of people. (B) The expression of FAM129A and RNF24 in granulocytes in different groups. (C) The expression of FAM129A and RNF24 in monocytles in different groups. (D)The expression of protein of FAM129A and RNF24 in granulocytes in different groups (E) Quantitative statistical results of protein expression. (F) The expression of protein of FAM129A and RNF24 in monocytles in different groups (G) Quantitative statistical results of protein expression. (*p < 0.05, **p < 0.01, ***p < 0.001, ns, *p* > 0.05).

**FIGURE 6:**
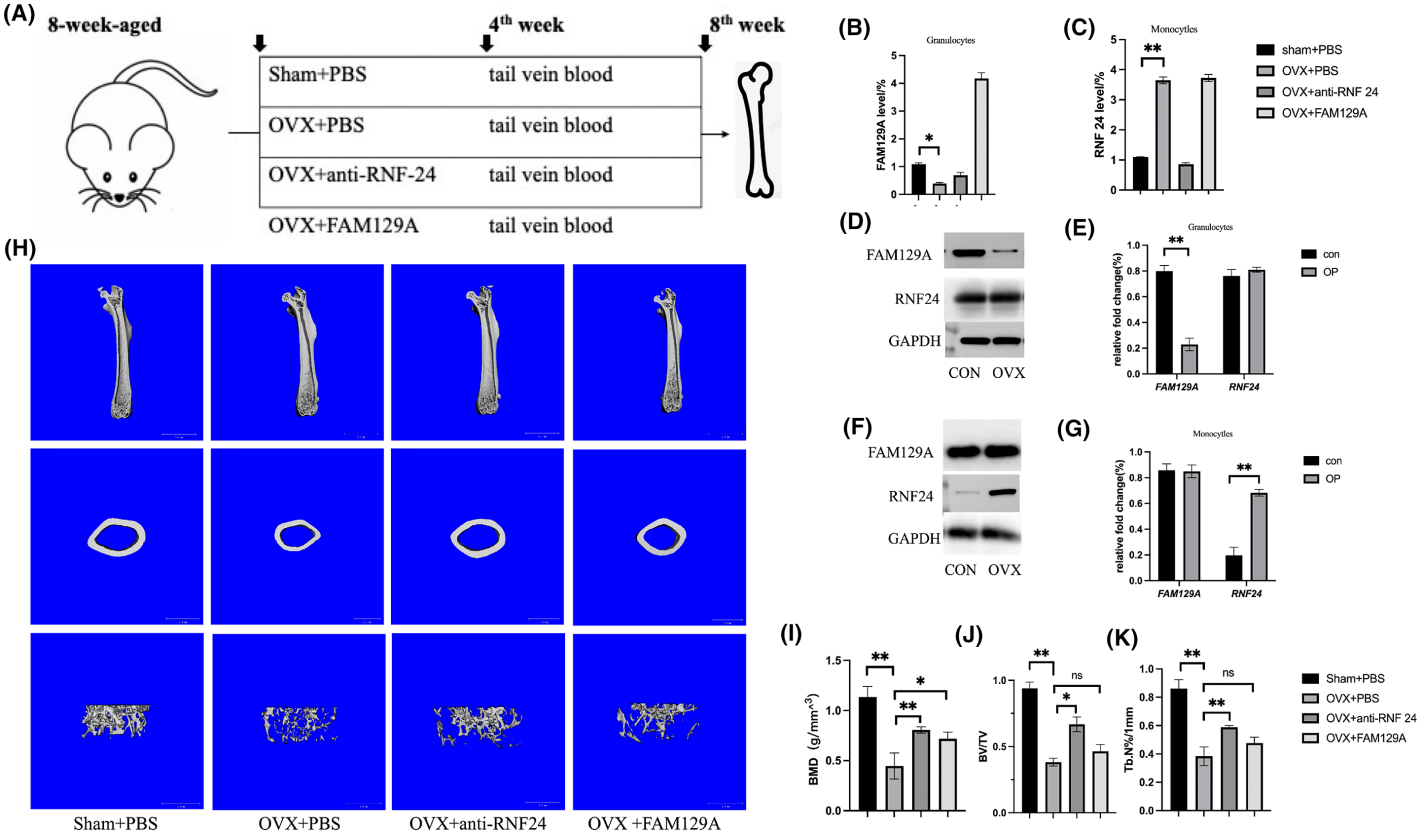
Analysis of the expression of FAM129A and RNF24 via mouse specimens. (A) Construction of different groups of mice and obtaining of peripheral blood and femur samples. (B) Expression of FAM129A in mice granulocytes of different groups. (C) Expression of RNF24 in mice granulocytes of different groups. (D) The expression of protein of FAM129A and RNF24 in granulocytes in different groups (E) Quantitative statistical results of protein expression. (F) The expression of protein of FAM129A and RNF24 in monocytles in different groups (G) Quantitative statistical results of protein expression. (H) Representative micro‐CT results of different groups. (I) Bone mineral density (BMD) of the micro‐CT results. (J) Bone volume/tissue volume (BV/TV) of the micro‐CT results for different groups. (K) Trabecular number (Tn.N) of the micro‐CT results for different groups. (*p < 0.05, **p < 0.01, ns, *p* > 0.05).

Human venous blood samples were obtained from 12 volunteers, and granulocyte and monocyte populations were obtained by gradient centrifugation (Figure 5A). As in the previous analysis (Figure 1B, C), neutrophils, myelocytes and BM occupy the top three in the grouping, and the neutrophil and myelocyte populations have a tendency to overlap, which is difficult to distinguish. Therefore, comprehensive detection of target genes in granulocytes can better verify the analysis results than neutrophils. qRT‐PCR results showed that the expression of FAM129A in granulocytes was significantly decreased in OP patients (Figure 5B), while the expression of RNF24 in monocytes was significantly increased (Figure 5C). Western‐blot was used to detect the protein expression of the target gene in neutrophils and monocytes. The results showed that FAM29A expression was significantly decreased in neutrophils, and RNF24 expression was significantly increased in monocytes (Figure 5D–G). Blood samples were obtained from mice tail vein after 4 weeks and femur samples were obtained after 8 weeks (Figure 6A). Subsequently, the blood from tail vein of the mice was separated by gradient centrifugation to obtain granulocytes and monocytes. qRT‐PCR results showed that the expression of FAM129A in granulocytes was significantly decreased (Figure 6B, D, E), and the expression of RNF24 in monocytes was significantly increased in osteoporotic mice (Figure 6C, F, G). Micro‐CT results suggested that the addition of FAM29A and anti‐RNF24 could significantly delay the progression of OP (Representative Figure 6H show the micro‐CT results) and improve bone mineral density (BMD) (Figure 6I), while anti‐RNF24 treatment could also increase Bone volume/tissue volume (BV/TV) (Figure 6J) and trabecular number (Tn.N) (Figure 6K).

## 4 DISCUSSION

Under the action of matrix proteins secreted by osteocytes (e.g. bone matrix proteins), focal adhesion can effectively bind osteocytes to the matrix, and play the functions of different bone cell types such as bone morphocytes, osteoblasts and bone resorptive cells.^24^ In OP patients, impairment of focal adhesion of osteocytes leads to an acceleration of the bone loss process. For example, focal adhesion damage of osteoblasts can prevent them from releasing bone matrix proteins normally and affect the bone construction function. The loss of focal adhesion of osteoclasts will lead to their inability to bind to the matrix and move normally during bone resorption, thereby accelerating bone loss.^25^ Currently, there is a lack of genetic‐level research on focal adhesion in OP. In this study, we utilized single‐cell and RNA‐seq data to identify relevant diagnostic genes for OP and evaluate their diagnostic value. Through differential gene identification and WGCNA coexpression network analysis, we identified two hub genes (FAM129 and RNF24) related to OP. Moreover, FAM129A in granulocytes and RNF24 in monocytes were found to be significantly different in the progression of OP by detection of human and mouse blood samples. Furthermore, it was preliminarily verified that RNF24 inhibition and FAM129A supplementation could significantly inhibit the progression of OP in a mouse model Figure S6.

In the first place, 30 candidate hub genes were authenticated based on the relevance analysis between two DE‐FARGs (FLNC and MAPK8 were acquired by overlapping 318 DEGs between OP and NC specimens, 489 key genes from WGCNA and 199 FARGs) and 131 common marker genes (131 common marker genes were filtered by overlapping 382 marker genes in neutrophils and 184 marker genes in neutrophil‐1). Moreover, these 30 candidate genes were highly correlated with focal adhesions. Further analysis revealed that these candidate genes were involved in the regulation of differentiation and cellular response to epidermal growth factor, as well as corticosteroid stimulus, all of which are important pathways related to the progression of OP. In particular, epidermal growth factor was found to induce the proliferation and differentiation of stem cells, leading to an increase in the number of granulocytes (inflammatory cells) and monocytes (precursors of osteoclasts). Meanwhile, cortisol was shown to inhibit osteoblast function, ultimately resulting in osteocyte apoptosis and bone loss.^26^, ^27^ Notably, these findings were consistent with the results of KEGG enrichment analysis.

Through in‐depth analysis of 30 candidate genes using different approaches, FAM129A and RNF24 were found to have potential predictive and diagnostic value for OP. Scholars have discovered that Family with Sequence Similarity 129 Member A (FAM129A) is a novel direct target gene of ATF4‐C/EBPβ, which is activated upon Endoplasmic Reticulum Stress (ER stress).^25^ Through consistent upregulation of the proapoptotic protein Bax and downregulation of the antiapoptotic protein Bcl‐2 by activating CCAAT/enhancer‐binding protein (C/EBP) homologous protein (CHOP), FAM129A ultimately induces the release of mitochondrial cytochrome C, formation of apoptotic bodies and cleavage of caspase‐3, leading to the induction of apoptosis.^26^ Single‐gene GSEA results suggest that the role of FAM129A in AP is related to the PPAR signalling pathway. Hence, we speculated that FAM129A regulate granulocyte apoptosis through PPAR/ATF4‐C/EBPβ, thus providing new insights into the underlying mechanisms of granulocyte apoptosis. RNF24 is a membrane protein that interacts with transient receptor potential channel (TRPC) proteins and regulates cell growth, differentiation, apoptosis and other biological processes by ubiquitinating target proteins.^28^ Recent studies have demonstrated the crucial role of RNF24 in various tumours.^29^ In lung cancer, the expression level of RNF24 is significantly upregulated, and it is involved in the regulation of the NF‐κB signalling pathway, promoting the proliferation and invasion of tumour cells. In breast cancer, RNF24 acts in conjunction with HER2 protein to accelerate the ubiquitination and degradation of HER2, thereby inhibiting the proliferation and invasion of tumour cells. Moreover, RNF24 regulates synaptic morphology and plasticity in the vertebrate nervous system, affecting neuronal information transmission and signal transduction. Despite the existing literature suggesting its involvement in spliceosome,^29^ neuroactive ligand–receptor interaction and actin cytoskeleton functions in different cells, research in osteocyte and the progression of OP remains rare. However, our findings suggest that RNF24, as a TRPC, may accelerate the progression of OP by regulating the intracellular calcium concentrations ([Ca^2+^] _i_),^30^ resulting in the activation of NF‐κB signalling pathways, which thereby affecting osteoclast adhesion and bone resorption.

To explore the regulatory relationships of hub genes in OP, we constructed the network based on hub genes, miRNAs, lncRNAs and TFs. The regulatory network of TF‐hub gene revealed that FAM129A and RNF24 could be regulated by POU2F2 and FOXC1. POU2F2 was shown to be an important transcription factor during the differentiation of MSCS into osteoblasts,^31^ and FOXC1 was considered to be an important gene for bone marrow stem cell migration and adhesion to cortical bone.^32^, ^33^ This regulatory network provides a direction for future research into the role and regulatory mechanisms of FAM129A and RNF24 in OP.

To predict potential effective treatments for OP, we leveraged the CTD database to identify therapeutic drugs. However, the role of the predicted drugs in OP still requires further exploration as potential therapeutic targets.

In summary, our study integrated single‐cell and RNA‐seq data using bioinformatics methods to explore the role of focal adhesion‐related genes (FARGs) in OP. These findings have clinical significance for the diagnosis and treatment of OP.

## AUTHOR CONTRIBUTIONS

**Xiaojian Shi:** Conceptualization (lead); project administration (lead); resources (lead); supervision (lead); writing – review and editing (lead). **Qiang Fu:** Data curation (equal); formal analysis (equal); software (equal). **Jianyu Mao:** Validation (equal). **Jiajie Yang:** Validation (equal). **Ye Chen:** Validation (equal). **Jiajia Lu:** Conceptualization (equal); data curation (equal); formal analysis (equal); investigation (equal); methodology (equal); software (equal); writing – original draft (equal). **Aimin Chen:** Validation (equal). **Nan Lu:** Data curation (equal); funding acquisition (equal); methodology (equal); resources (equal).

## FUNDING INFORMATIOM

This research was funded by Sailing Program of Naval Medical University, and Program of Shanghai Hongkou District Health Commission (grant No. 2202‐27), and Scientific Research Project of Nantong Health Commission (grant No. MS2023115).

## CONFLICT OF INTEREST STATEMENT

The authors declare no conflict of interest.

## INFORMED CONSENT STATEMENT

Informed consent was obtained from all subjects involved in the study. Written informed consent has been obtained from the patients to publish this paper.

## Supporting information


Figure S1.

Figure S2.

Figure S3.

Figure S4.

Figure S5.

Figure S6.

Table S1.

Table S2.

Table S3.

Table S4.

Table S5.

Table S6.

## Data Availability

Data are contained within the article or supplementary material. The data presented in this study are available on request from the corresponding author.
