# Correction to ‘Integration of Single‐Cell and RNA‐Seq Data to Explore the Role of Focal Adhesion‐Related Genes in Osteoporosis’

**DOI:** 10.1111/jcmm.71291

**Published:** 2026-08-14

X. Shi, Q. Fu, J. Mao, et al., “Integration of Single‐Cell and RNA‐Seq Data to Explore the Role of Focal Adhesion‐Related Genes in Osteoporosis,” *Journal of Cellular and Molecular Medicine* 28 (2024): e18271, https://doi.org/10.1111/jcmm.18271.

In Xiaojian Shi et al., the Western blot (WB) band of RNF24 in Figure 6D was presented incorrectly due to a technical error during image preparation by the authors. The corrected image is shown below. The authors confirm all results and conclusions of this article remain unchanged.

We apologize for this error.

**FIGURE 6:**
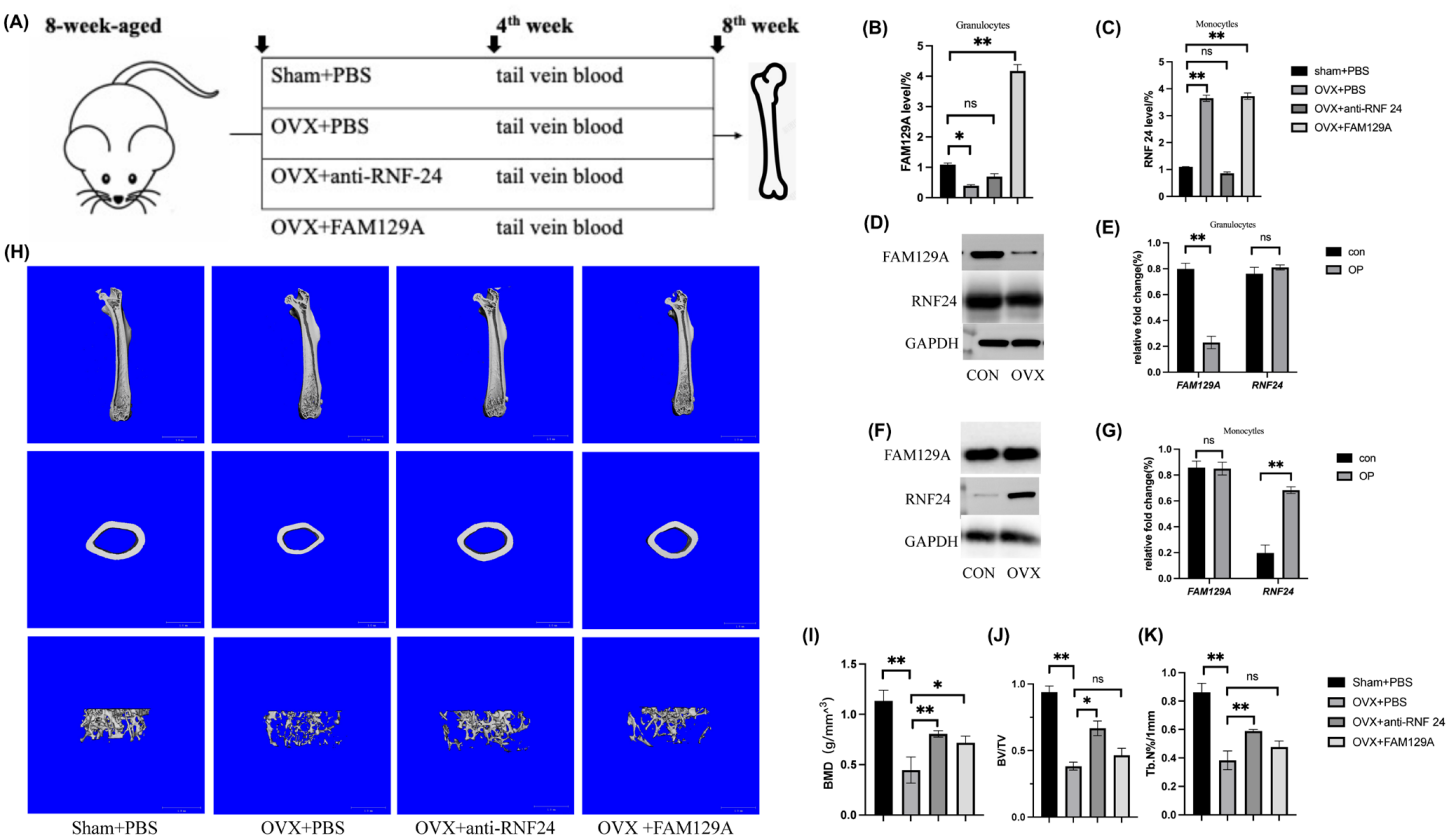
Analysis of the expression of FAM129A and RNF24 via mouse specimens. (A) Construction of different groups of mice and obtaining of peripheral blood and femur samples. (B) Expression of FAM129A in mice granulocytes of different groups. (C) Expression of RNF24 in mice granulocytes of different groups. (D) The expression of protein of FAM129A and RNF24 in granulocytes in different groups. (E) Quantitative statistical results of protein expression. (F) The expression of protein of FAM129A and RNF24 in monocytes in different groups. (G) Quantitative statistical results of protein expression. (H) Representative micro‐CT results of different groups. (I) Bone mineral density (BMD) of the micro‐CT results. (J) Bone volume/tissue volume (BV/TV) of the micro‐CT results for different groups. (K) Trabecular number (Tn.N) of the micro‐CT results for different groups (*p < 0.05, **p < 0.01, ns, *p* > 0.05).

