# Supplementary material for: Integration of single‐cell and RNA‐seq data to explore the role of focal adhesion‐related genes in osteoporosis
Source: J Cell Mol Med. 2024 Mar 27;28(8):e18271. doi: 10.1111/jcmm.18271 (PMC10967139; doi:10.1111/jcmm.18271)
Supplement: Supplementary file 1 — Figure S1. [file JCMM-28-e18271-s003.zip › Figure S1 caption.docx]

Figure S1. Quality control of single-cell sequencing data. (A) Distribution of nFeature_RNA, nCount_RNA and percent.mt before quality control. (B) Distribution of nFeature_RNA, nCount_RNA and percent.mt after quality control. (C) Identification of 2000 hypervariable genes. (D) PCA dimensionality reduction analysis. (E) The JackStraw method to screen the alternative dimensions. (F) The JackStraw method to screen the alternative dimensions.
