# Supplementary figures and images for: Integration of single‐cell and RNA‐seq data to explore the role of focal adhesion‐related genes in osteoporosis
Source: J Cell Mol Med. 2024 Mar 27;28(8):e18271. doi: 10.1111/jcmm.18271 (PMC10967139; doi:10.1111/jcmm.18271)

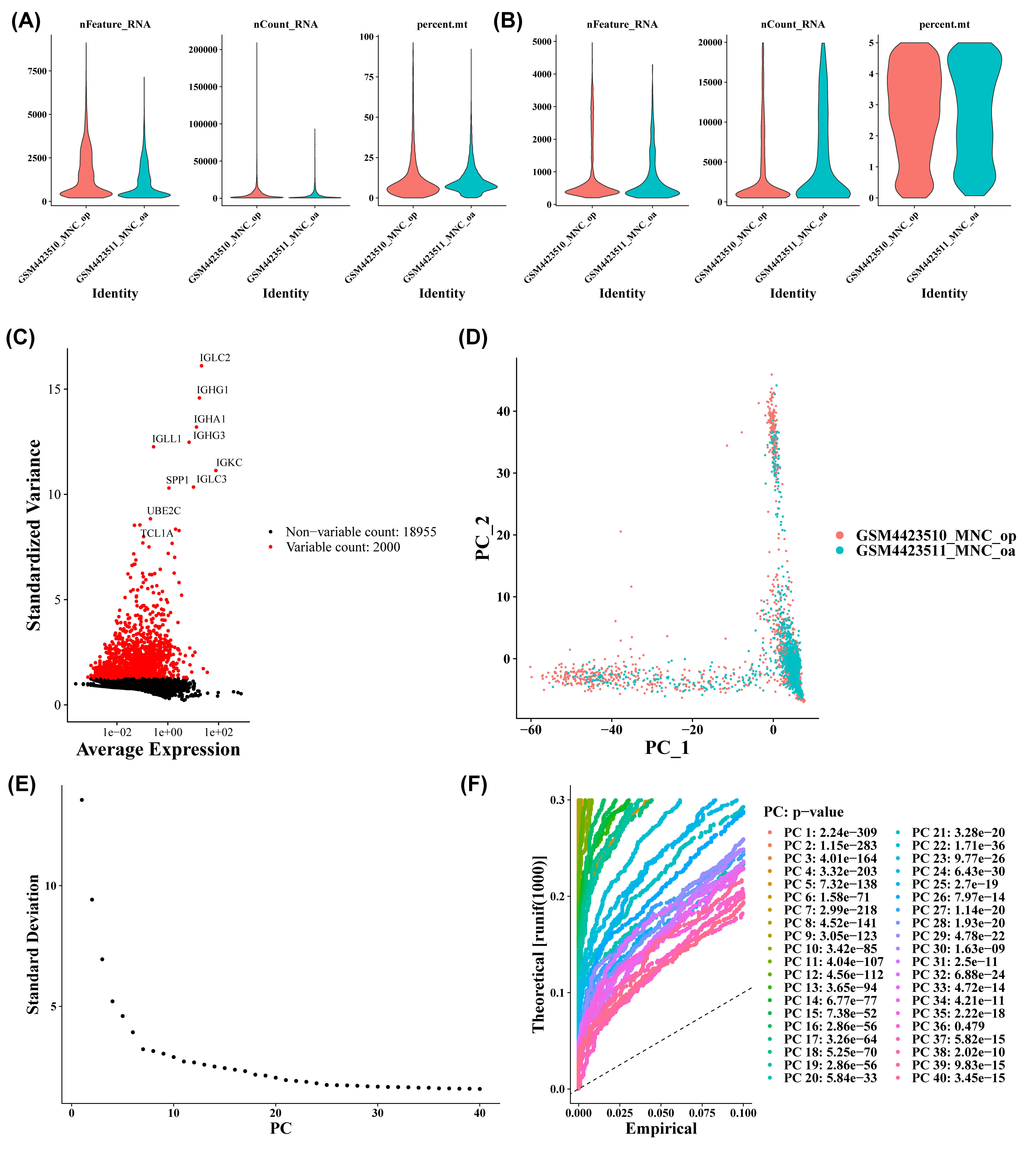

Supplement: Supplementary file 1 — Figure S1. [file JCMM-28-e18271-s003.zip › Figure S1.tif]

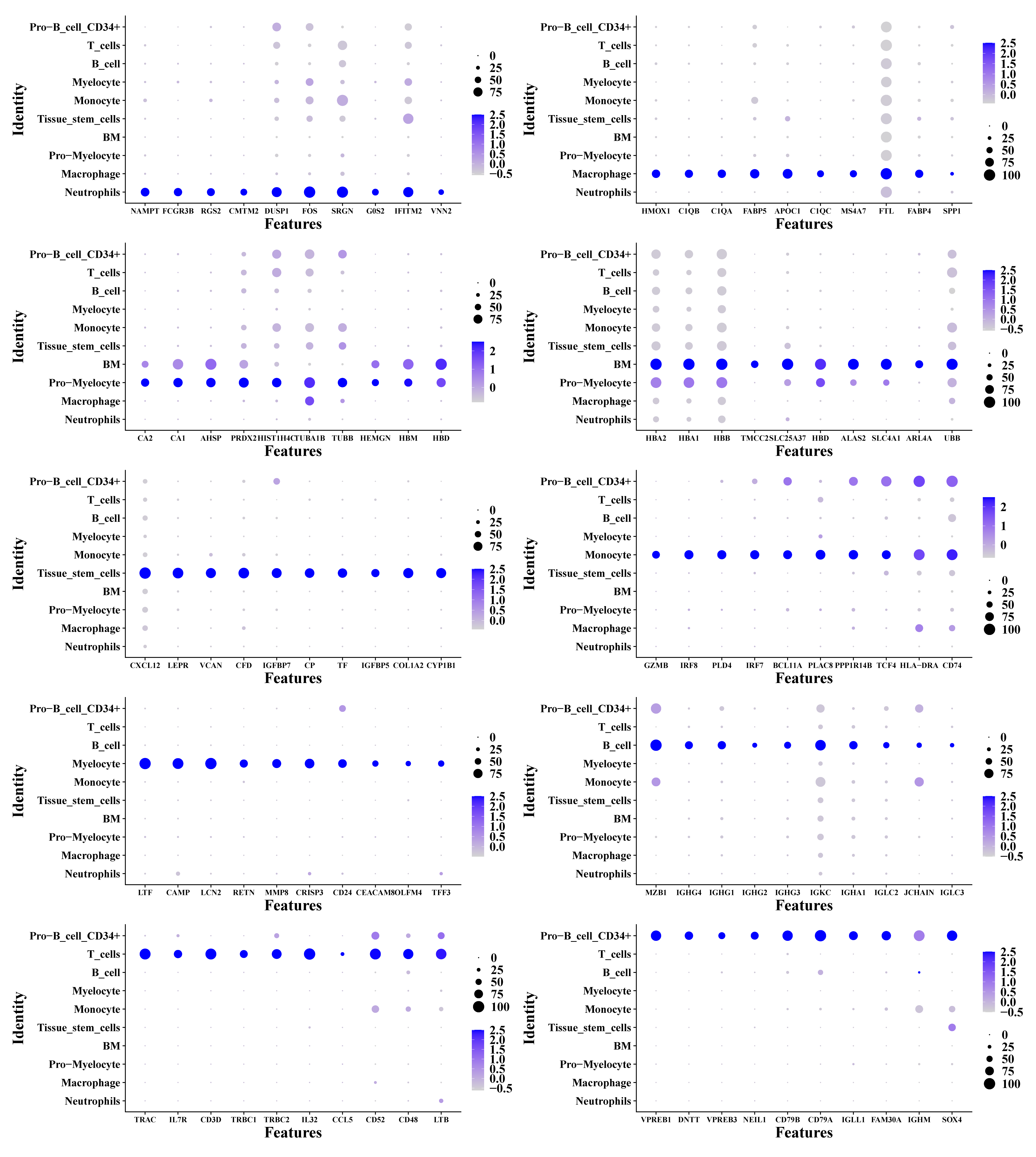

Supplement: Supplementary file 2 — Figure S2. [file JCMM-28-e18271-s002.zip › Figure S2.tif]

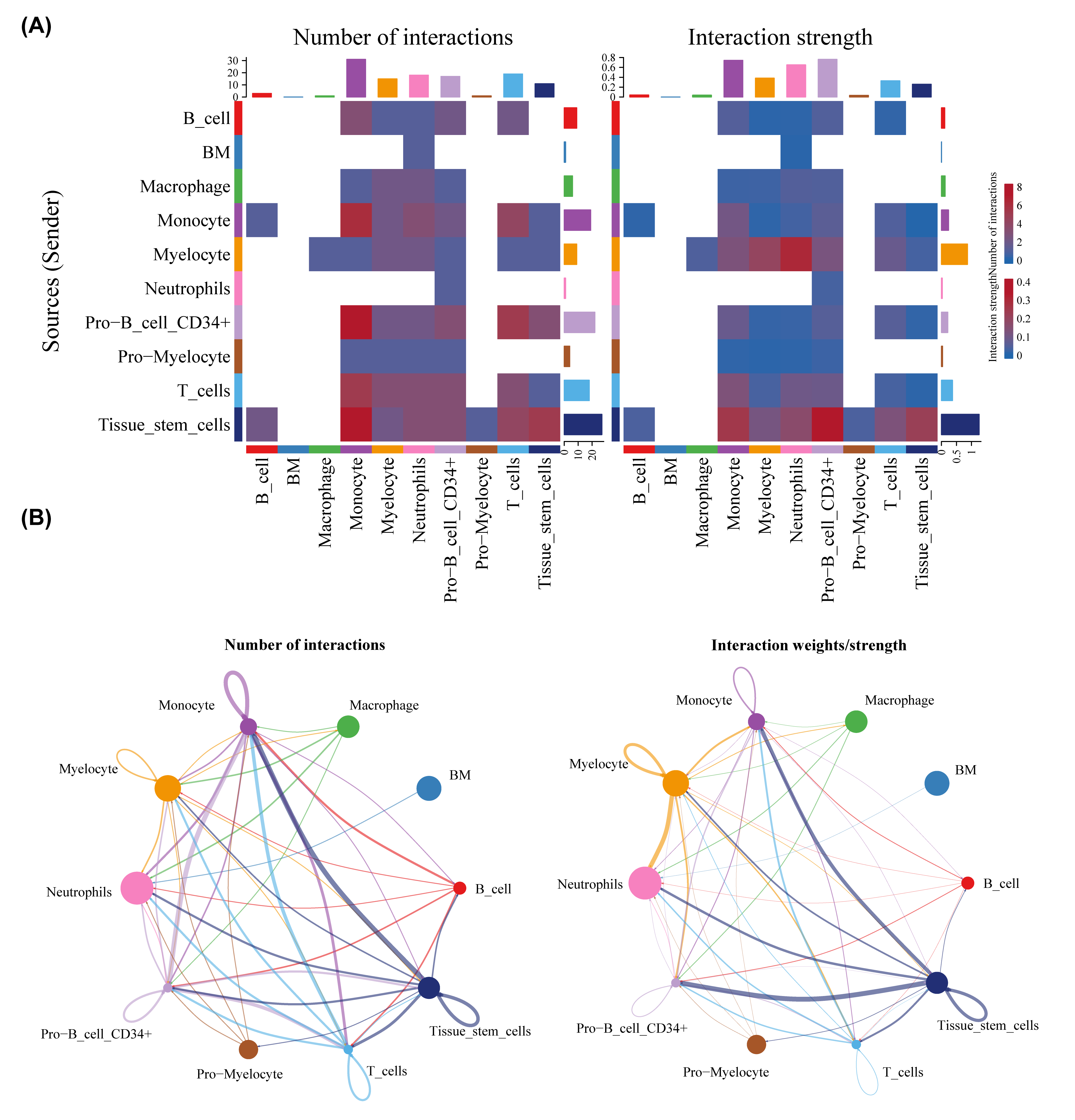

Supplement: Supplementary file 3 — Figure S3. [file JCMM-28-e18271-s009.zip › Figure S3.tif]

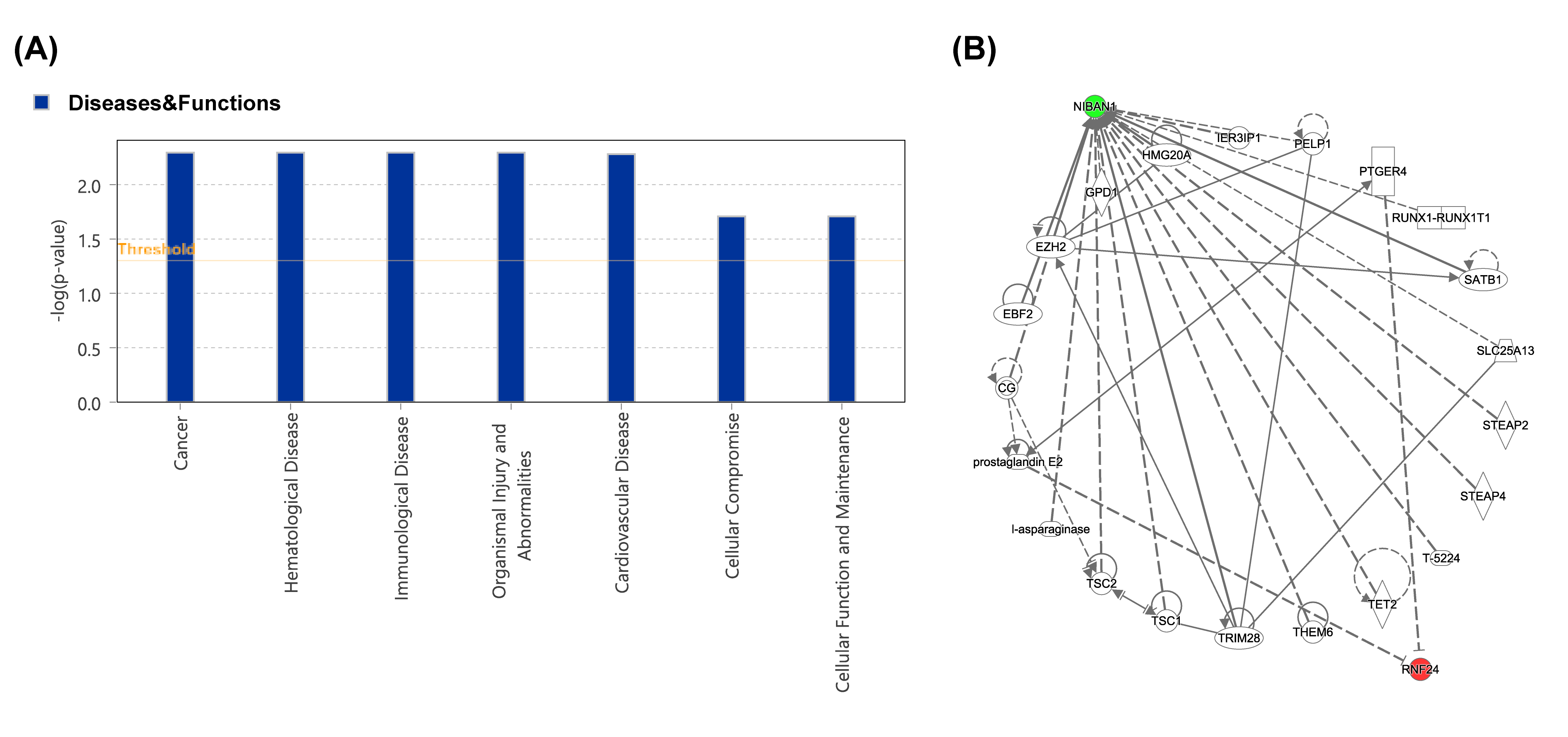

Supplement: Supplementary file 4 — Figure S4. [file JCMM-28-e18271-s011.zip › Figure S4.tif]

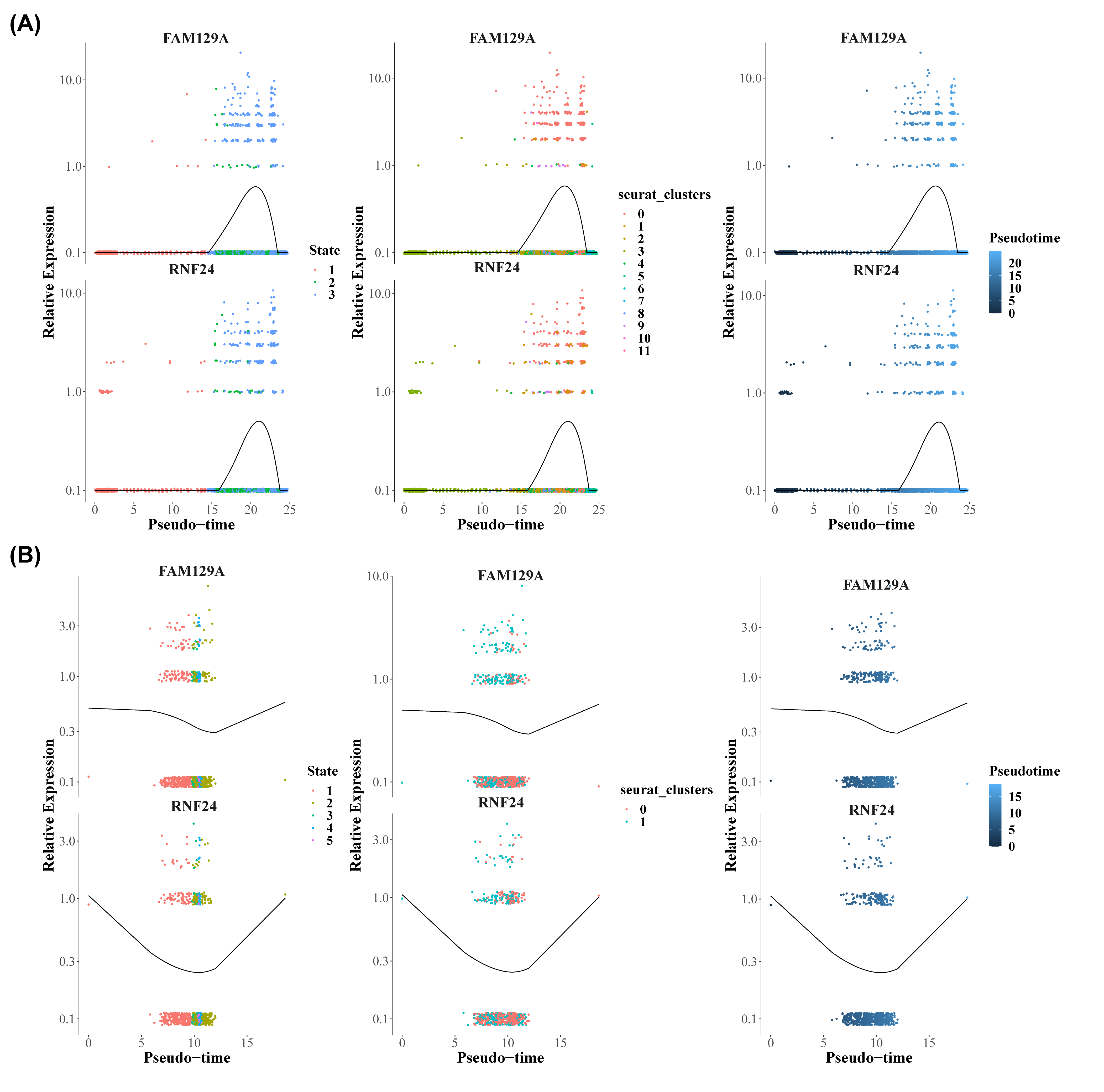

Supplement: Supplementary file 5 — Figure S5. [file JCMM-28-e18271-s012.zip › Figure S5.tif]
