# Supplementary material for: Integration of single‐cell and RNA‐seq data to explore the role of focal adhesion‐related genes in osteoporosis
Source: J Cell Mol Med. 2024 Mar 27;28(8):e18271. doi: 10.1111/jcmm.18271 (PMC10967139; doi:10.1111/jcmm.18271)
Supplement: Supplementary file 2 — Figure S2. [file JCMM-28-e18271-s002.zip › Figure S2 caption.docx]

Figure S2. (A) The expression of top10 marker genes in 10 cell subpopulations for each cell type.
