# Supplementary material for: Integration of single‐cell and RNA‐seq data to explore the role of focal adhesion‐related genes in osteoporosis
Source: J Cell Mol Med. 2024 Mar 27;28(8):e18271. doi: 10.1111/jcmm.18271 (PMC10967139; doi:10.1111/jcmm.18271)
Supplement: Supplementary file 3 — Figure S3. [file JCMM-28-e18271-s009.zip › Figure S3 caption.docx]

Figure S3. Cell communication analysis of 10 cell types. (A) Heat map of cell communication. (B) Circle diagram of cell communication.
