# Supplementary material for: Integration of single‐cell and RNA‐seq data to explore the role of focal adhesion‐related genes in osteoporosis
Source: J Cell Mol Med. 2024 Mar 27;28(8):e18271. doi: 10.1111/jcmm.18271 (PMC10967139; doi:10.1111/jcmm.18271)
Supplement: Supplementary file 4 — Figure S4. [file JCMM-28-e18271-s011.zip › Figure S4 caption.docx]

Figure S4. IPA analysis of 2 hub genes. (A) Diseases and functions analyses of 2 hub genes (B) The regulation of 2 hub genes.
