# Supplementary material for: Integration of single‐cell and RNA‐seq data to explore the role of focal adhesion‐related genes in osteoporosis
Source: J Cell Mol Med. 2024 Mar 27;28(8):e18271. doi: 10.1111/jcmm.18271 (PMC10967139; doi:10.1111/jcmm.18271)
Supplement: Supplementary file 5 — Figure S5. [file JCMM-28-e18271-s012.zip › Figure S5 caption.docx]

Figure S5. The relative expression of FAM129A and RNF24 in different state of differentiation. (A) FAM129A and RNF24 expression at different states. (B) The expression of hub genes during the differentiation of neutrophils.
